# Effect of thermometry on the prevention of diabetic foot ulcers: a systematic review with meta-analysis*

**DOI:** 10.1590/1518-8345.5663.3525

**Published:** 2022-05-16

**Authors:** Açucena Leal de Araújo, Francisca Diana da Silva Negreiros, Raquel Sampaio Florêncio, Shérida Karanini Paz de Oliveira, Ana Roberta Vilarouca da Silva, Thereza Maria Magalhães Moreira

**Affiliations:** 1 Universidade Estadual do Ceará, Fortaleza, CE, Brasil.; 2 Bolsista da Coordenação de Aperfeiçoamento de Pessoal de Nível Superior (CAPES), Brasil.; 3 Universidade Federal do Ceará, Fortaleza, CE, Brasil.; 4 Universidade Federal do Piauí, Picos, PI, Brasil.; 5 Bolsista do Conselho Nacional de Desenvolvimento Científico e Tecnológico (CNPq), Brasil.

**Keywords:** Diabetes Mellitus, Thermometry, Diabetic Foot, Prevention, Nursing, Systematic Review, Diabetes Mellitus, Termometria, Pé Diabético, Prevenção de Doenças, Enfermagem, Revisão Sistemática, Diabetes Mellitus, Termometría, Pie Diabético, Prevención de Enfermedades, Enfermería, Revisión Sistemática

## Abstract

**Objective:**

to analyze the effect of cutaneous foot thermometry in people with Diabetes Mellitus, compared with the standard prevention of foot ulcers adopted in these patients.

**Method:**

a systematic review with meta-analysis. Protocol registered with PROSPERO (CRD42020202686). The recommendations of the Preferred Reporting Items for Systematic Reviews and Meta-Analyses (PRISMA) were followed. The search was performed in the following data sources: SCOPUS, Web of Science, MEDLINE via PubMed, MEDLINE via EBSCO, MEDLINE via *Biblioteca Virtual em Saúde*, Embase, CINAHL, Cochrane Library, LILACS via *Biblioteca Virtual em Saúde*, Google Scholar, *Biblioteca Digital Brasileira de Teses e Dissertações, Catálogo de Teses & Dissertações-Capes*, Open Grey and ProQuest Dissertations and Theses. The risk of bias was assessed by the Cochrane Collaboration Risk of Bias Tool (RoB 2), the meta-analysis was performed in the Review Manager 5.4 software and the Certainty of evidence in the Grading of Recommendations Assessment, Development and Evaluation system.

**Results:**

of the 670 records, five articles were eligible. The meta-analysis was calculated for the prevention of the incidence of diabetic foot ulcers outcome, with effect summarization (RR 0.53; 95%CI 0.29-0.96; p=0.02), with certainty of moderate evidence.

**Conclusion:**

thermometry showed a protective effect on the incidence of diabetic foot ulcers when compared to standard foot care.

## Resumo

**Objetivo:**

analisar o efeito da termometria podálica cutânea em pessoas com diabetes mellitus, comparado com prevenção padrão de úlceras podálicas adotada nesses pacientes.

**Método:**

revisão sistemática com metanálise. Protocolo registrado na PROSPERO (CRD42020202686). Foram seguidas as recomendações do *Preferred Reporting Items for Systematic Reviews and Meta-Analyses* (PRISMA). Busca realizada nas fontes de dados: SCOPUS, *Web of Science*, MEDLINE via PubMed, MEDLINE via EBSCO, MEDLINE via Biblioteca Virtual em Saúde, Embase, CINAHL, *Cochrane Library*, LILACS via Biblioteca Virtual em Saúde, *Google Scholar*, Biblioteca Digital Brasileira de Teses e Dissertações, Catálogo de Teses & Dissertações-Capes, *Open Grey* e *ProQuest Dissertations and Theses*. O risco de viés foi avaliado pela *Cochrane Collaboration Risk of Bias Tool* (RoB 2), a metanálise no *software Review Manager* 5.4 e a Certeza da evidência no sistema *Grading of Recommendations Assessment, Development and Evaluation*.

**Resultados:**

dos 670 registros, cinco artigos eram elegíveis. A metanálise foi calculada para o desfecho prevenção da incidência de úlceras de pé diabético, com sumarização de efeito (RR 0,53; IC95% 0,29, 0,96; p=0,02), com certeza da evidência moderada.

**Conclusão:**

a termometria mostrou efeito protetor na incidência de úlceras de pé diabético, se comparada ao cuidado podálico padrão.

## Resumen

**Objetivo:**

analizar el efecto de la termometría cutánea del pie en personas con diabetes mellitus, en comparación con la prevención estándar de las úlceras del pie adoptada en estos pacientes.

**Método:**

revisión sistemática con metaanálisis. Protocolo registrado en PROSPERO (CRD42020202686). Se siguieron las recomendaciones de *Preferred Reporting Items for Systematic Reviews and Meta-Analyses* (PRISMA). La búsqueda se realizó en las fuentes de datos: SCOPUS, *Web of Science*, MEDLINE a través de PubMed, MEDLINE a través de EBSCO, MEDLINE a través de la Biblioteca Virtual en Salud, Embase, CINAHL, *Cochrane Library*, LILACS a través de la Biblioteca Virtual en Salud, *Google Scholar*, Biblioteca Digital Brasileña de Tesis y Disertaciones, Catálogo de Tesis y Disertaciones-Capes, *Open Grey* y *ProQuest Dissertations and Theses*. El riesgo de sesgo se evaluó mediante la *Cochrane Collaboration Risk of Bias Tool* (RoB 2), el metaanálisis se realizó con el *software Review Manager* 5.4 y la certeza de la evidencia se evaluó mediante el sistema *Grading of Recommendations Assessment, Development and Evaluation*.

**Resultados:**

de los 670 registros, cinco artículos fueron elegibles. El metaanálisis se calculó para el resultado prevención de la incidencia de úlceras del pie diabético, con sumarización del efecto (RR 0,53; IC95% 0,29, 0,96; p=0,02), con certeza de evidencia moderada.

**Conclusión:**

la termometría mostró un efecto protector sobre la incidencia de úlceras del pie diabético en comparación con el cuidado estándar del pie.

**Highlights**

(1) Diabetic foot ulcers are preceded by an increase in local dermal temperature.

(2) Thermometry can assist in the early identification of inflammation/ulceration.

(3) Temperature measurements can be easily taken by the patients/family members.

(4) Thermometry can contribute to reducing the burden on the health services.

(5) The efficacy of thermometry is supported by the certainty of moderate evidence.

## Introduction

The current approaches adopted by health services in the prevention and early treatment of Diabetic Foot Ulcers (DFUs) are multiple and varied. Screening and education in health for the patient, family and health professionals are relevant pillars^1^. However, efforts to prevent DFU remain a challenge and demand high costs for global public health^2^ ^-^ ^3^, raising the need for new preventive approaches.

DFUs are preceded by an increase in local dermal temperature by inflammation and enzymatic tissue autolysis resulting from pressure-activity imbalance, added to repetitive stress, neuropathic sensory loss and biomechanical abnormalities^4^. A temperature difference of 2.2ºC between the same site on both feet implies a risk for imminent ulcer/inflammation^5^ ^-^ ^6^. However, clinical signs of inflammation are subtle for detection by patients or even by trained health professionals^7^. Although many signs of inflammation are difficult to be objectively assessed, temperature can be easily measured. 

The traditional method of assessing foot temperature is palpation with the back of the hand. However, with this method, humans are only able to discriminate temperature differences greater than 2ºC. Thus, skin thermometry emerges as a promising tool for identifying inflammation, providing early signs to prevent DFU incidence and reduce serious complications, such as high morbidity, frequent hospitalizations, lower limb amputation and deaths^8^ ^-^ ^9^. It is the method most used by the scientific community and patients, as it involves the use of a thermometer, a low-cost and easy to apply instrument^10^. As a result, the patients can modify their activity, measuring skin temperature, as well as dosing their insulin and checking their blood glucose^11^.

A study reported an association between increased local temperature and localized pressure, causing tissue damage^12^. A number of researchers used thermometry as a tool to diagnose occult neuropathic fractures in patients with diabetes^13^. Temperature assessment is a useful technique to identify patients at risk for ulceration^14^. Similar findings were identified with a handheld infrared thermometer in patients with asymptomatic sensory neuropathy, neuropathic foot ulcers and patients with neuropathic fractures (Charcot arthropathy)^15^.

Foot temperature varies with the patient’s activity level and environment. The reference is a corresponding area on the contralateral foot. It is noteworthy that the temperatures of these areas do not differ by more than 1ºC^5^ ^-^ ^6^ and a number of studies suggest that differences ≥ 2.2ºC in temperature can be considered a risk for ulceration^11^ ^,^ ^16^ ^-^ ^18^.

*Worldwide, three Randomized Controlled Clinical Trials (RCTs) tested self-monitoring of foot skin temperature in people with Diabetes Mellitus and their risk for ulceration, via skin infrared thermometry as a warning sign of impending ulcer. These studies showed a significant reduction in the incidence of new foot ulcers* ^*11*^ ^*,*^ ^*16*^ ^*-*^ ^*17*^ *.*

Previous systematic reviews have analyzed the effect of several interventions to prevent diabetic foot ulcers, including the use of thermal foot monitoring^19^ ^-^ ^21^. Although reviews on the subject matter were identified, failures to provide reliable numerical summaries of effects were observed due to limitations in the quality of the individual studies. In addition to that, new evidence has emerged since then. Thus, a comprehensive evaluation of randomized controlled trials was necessary to allow us to make the best use of the currently available evidence.

This systematic review with meta-analysis aimed at analyzing the effect of cutaneous foot thermometry in people with Diabetes Mellitus (DM), when compared to the standard prevention of foot ulcers adopted in these patients.

## Method

### Protocol and registration

This is a systematic literature review, according to the criteria of the *Prefe*rred Reporting Items for Systematic Reviews and Meta-Analyses (PRISMA)^22^. This type of study summarizes diverse evidence from primary studies conducted to answer a specific research question. It uses a comprehensive, impartial and reproducible literature review process, and locates, evaluates and synthesizes the set of evidence from the scientific studies to obtain an overview and reliable estimate of the intervention’s effect^23^.

This review had its protocol previously published on York University’s International Prospective Register of Systematic Reviews (PROSPERO) platform, with registration number CRD42020202686, obtained on September 4^th^, 2020.

### Research question and eligibility criteria

The Population, Intervention, Control and Outcome (PICO) strategy was used for data research^24^. This systematic review with meta-analysis focused on participants (P) diagnosed with Diabetes Mellitus, with or without risk of developing diabetic foot ulcer. The use of cutaneous thermometry devices to assess foot temperature was arranged as an intervention (I). The use of standard foot care (therapeutic footwear, diabetic foot education, regular foot assessment by health professionals, and foot self-care) was provided as a comparison (C). And the outcomes (O) evaluated were studies that included the prevention of the incidence of diabetic foot ulcers outcome. 

From this, the research problem was outlined: Which is the effect of cutaneous foot thermometry on people with DM, when compared to the standard prevention of foot ulcers adopted in these patients?

The studies were included when they met the following criteria: DM diagnosis, age ≥ 18 years old, clinical trial with intervention group with thermometry in the prevention of Diabetic Foot Ulcers (DFUs) and control group with standard health care. No restrictions were adopted regarding language and year of publication, nor in relation to the risk for DFU (according to the stratification of the International Working Group on Diabetic Foot)^1^. We discarded publications with: study intervention with foot skin thermometry in people with and without DM; thermometry in people with simultaneous Diabetes Mellitus and active ulcer; study designs: cross-sectional, prospective and retrospective cohort, case-control, case reports or case series; types of publication: reviews, protocols, letters to the editor, congress abstracts, personal opinions, book chapters; unavailable in full.

### Search in data sources

The search was performed in the gray literature and in the databases on July 26^th^, 2020, without language or year restrictions. An update of the searches in the data sources was performed on November 21^st^, 2021. A librarian familiar with the health sciences was consulted when developing and conducting the research. 

The databases used were the following: SCOPUS, Web of Science, MEDLINE (Medical Literature Analysis and Retrieval System Online) via PubMed, MEDLINE (Medical Literature Analysis and Retrieval System Online) via EBSCO, MEDLINE (Medical Literature Analysis and Retrieval System Online) via *Biblioteca Virtual em Saúde* (BVS), Embase, CINAHL (Cumulative Index to Nursing and Allied Health Literature), Cochrane Library (The Cochrane Central Register of Controlled Trials - CENTRAL) and LILACS (*Literatura Latino-Americana e do Caribe em Ciências da Saúde*) via *Biblioteca Virtual em Saúde* (BVS). 

In order to reduce publication bias and identify as much relevant evidence as possible, the following gray literature databases were also consulted: Google Scholar, *Biblioteca Digital Brasileira de Teses e Dissertações* (BDTD), *Catálogo de Teses & Dissertações* - CAPES, Open Grey and ProQuest Dissertations and Theses (PQDT). 

Additionally, another search for the references of the studies included was necessary. An additional search was also carried out in consultation with “experts/specialists” in the researched area via https://www.researchgate.net/. They were invited to suggest relevant articles on the chosen topic. However, no answers were obtained.

### Search strategy

The search strategy was built using three controlled health vocabularies: Medical Subject Headings (MeSH), *Descritores em Ciências da Saúde* (DeCS) and EMTREE; together with natural language, in order to obtain a wide spectrum of results in different databases^25^. After the search terms have been defined, they were combined with the Boolean operators AND and OR. 

The following high-sensitivity search strategy was used for all databases: (“diabetes mellitus” OR “diabetic patient” OR diabetes OR diabetic) AND (“temperature measurement” OR “temperature monitoring” OR “temperature recording” OR “thermal measurement” OR “thermal monitoring” OR “thermal recording” OR “thermo-monitoring” OR “thermo-recording” OR “body temperature monitoring” OR thermometer OR thermometry OR thermogram OR thermomonitoring OR thermorecording OR thermomeasurement) AND (prevention OR prevent OR control OR prophylaxis OR “prevent ulceration” OR “ulcer prevention” OR “prophylactic treatment” OR “preventive therapy” OR “preventive measures” OR “disease prevention” OR “disease prophylaxis” OR “health protection” OR “preventive treatment” OR “prophylactic management” OR “prophylactic therapy” OR “prophylactic treatment”) AND (foot OR ulcer OR ulceration OR feet OR “foot ulceration” OR “foot ulcer” OR “foot complication” OR “diabetic foot” OR “diabetic feet”).

### Selection of the studies and extraction of the information

The research results in each database were imported into the Rayyan^®^ reference manager developed by the Qatar Computing Research Institute (QCRI), for organization of the studies, removal of duplicates, and selection and screening of the studies^26^. Two authors of the review independently examined the titles and abstracts of all references. Subsequently, the full texts of potentially eligible studies were independently assessed by the two reviewers to determine whether all inclusion criteria were met. In case of disagreements, the third author of the review was consulted.

The data from the studies selected for the final sample were independently extracted by the two reviewers and then compared. A standardized clinical form created in *Microsoft Excel* ^®^ was used and, finally, the data collected were compiled into a table. The data collected were the following: study characteristics (author, year, country, objective); population characteristics (total sample, gender, type of diabetes and mean age), characteristics of the intervention and control groups (place of sample recruitment, duration in months, number of participants in each group, description of the intervention and of the control), characteristics of the result (outcomes evaluated, main outcomes) and conclusion. Afterwards, the results of collection were compared, discussing what was really relevant with the third reviewer. If there was information that was lacking, ambiguous, incomplete or not described in the primary studies, efforts were made to contact the authors.

The Preferred Reporting Items for Systematic Reviews and Meta-Analyses (PRISMA) flowchart was used to document selection of the studies.

### Risk of bias analysis in the individual studies

The risk of bias assessment of the studies included was analyzed using the Cochrane Collaboration Risk of Bias Tool (RoB 2) for randomized studies^27^. RoB 2 includes judgments about random sequence generation, allocation concealment, participant and staff blinding, results evaluator blinding, incomplete results data, selective reporting, and other sources of bias. The studies were categorized into groups labeled as low risk of bias, uncertain risk of bias, or high risk of bias.

RoB 2 has the novelty of considering that it is not always possible to blind the participants. We should only lower the quality of the evidence if lack of blinding affects the interventions in an unbalanced way between the groups and if it affects outcomes^27^. Risk of bias was performed by two independent reviewers. In case of disagreements in the judgment of the two reviewers, an evaluation by the third reviewer was requested.

To summarize the risk of bias analysis, the Cochrane Collaborations’s Review Manager 5 (RevMan 5.4) tool was used and allowed for the elaboration of the risk of bias summary^28^. 

### Data synthesis

A descriptive synthesis of the characteristics of the studies was carried out. The quantitative synthesis of the data was performed in a meta-analysis of studies considered combinable and homogeneous in relation to interventions and results. Cochrane Collaboration’s Review Manager^®^5 (RevMan 5.4)^28^ was used to perform the meta-analysis, presented using a forest plot graph. The heterogeneity present in the studies was evaluated by the I2 statistical test of inconsistency indices, considering values of 0-30% for unimportant heterogeneity, 31-50% for moderate heterogeneity, 51-80% for substantial heterogeneity and 81-100% for considerable heterogeneity^29^. The results were presented with 95% Confidence Intervals (95%CI).

The random effect model was chosen^29^ to perform the meta-analysis. As a measure of effect, for binary outcomes (the result can be 1 of 2 possibilities) the Risk Ratio (RR, or relative risk) was used, considering in this study the occurrence or not of diabetic foot ulcer. As for the estimate of the effect of the individual studies, this was calculated using the Mantel-Haenszel statistical method, ideal for studies with small sample sizes.

### Evidence certainty classification

This research used Cochrane’s Grading of Recommendations Assessment, Development and Evaluation (GRADE) system to classify the certainty of the evidence^30^. The criteria evaluated were study design, risk of bias, inconsistency, indirect evidence, imprecision, and other considerations. The certainty of the evidence can be characterized as high, moderate, low or very low. The construction of a funnel chart to assess the presence of publication bias was waived because the few randomized clinical trials were less than ten. 

A table called “GRADE Evidence Profile” was created to summarize the findings of this evaluation. In this process, the GRADEpro GDT (Guideline Development Tool) online tool was used, which is freely accessible from http://www.gradepro.org.

## Results

### Characteristics of the studies included

**Figure 1:**
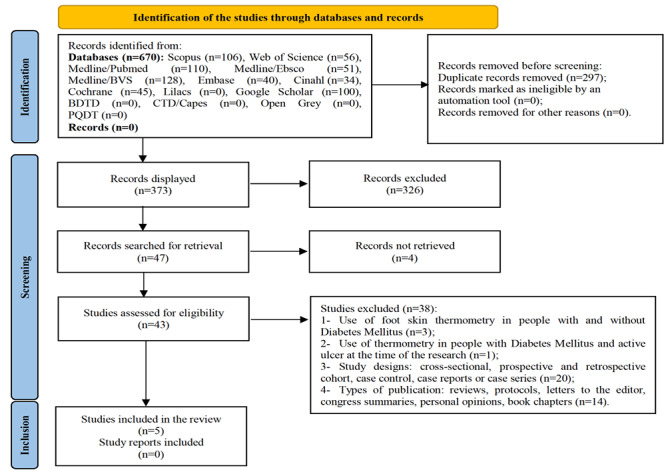
Search flowchart according to the PRISMA recommendations^22^

A total of 670 records were identified. After removing the duplicates, 373 were left for screening. After the titles and abstracts have been screened, 326 records were excluded for not meeting the inclusion criteria, leaving 47 potentially relevant studies. Of these, 4 records were not retrieved in full, leaving 43 for full-text screening. After full-reading, 38 did not meet the eligibility criteria, leaving 5 studies (Figure 1). 

The final result were five Randomized Clinical Trials (RCTs), with a total of 828 participants, conducted in the United States^11^ ^,^ ^16^ ^-^ ^17^, Holland^31^ and Norway^32^ and published in English between 2004 and 2021. The characteristics of the articles reveal groups consisting mostly of aged men, diagnosed with DM2, long-term Diabetes Mellitus and high risk stratification for ulceration 2/3^1^.

The studies had follow-ups from six to 18 months. All studies evaluated the use of thermometry at home as a preventive method for diabetic foot ulcers compared to standard health care. The thermometry device used in all interventions was a handheld infrared digital thermometer (TempTouch, Diabetica Solutions, San Antonio, Texas, USA). The controls used standard foot care (follow-up with health professionals, foot care education, use of therapeutic footwear). Figure 2 summarizes the characteristics of the studies included.

**Figure 2:** Characteristics of the RCTs included in the SR (n=5). Fortaleza, CE, Brazil, 2021

Author/ Year/ Country	Characteristics of the sample	Intervention Group/Control Group			Main results
Armstrong, et al.^16^ 2007. United States.	n=225 (IG*: 110, CG^†^: 115) Male: 96% Mean age: 69 years old DM2^‡^: 100% Mean time since DM diagnosis: 13 years Risk of ulcer: 2/3 (IWGDF^§^)	Duration: 18 months Outcomes: Proportion of patients in each group who developed foot ulcers	IG*: Handheld infrared thermometer and recording in diary	CG^†^: Therapeutic footwear, education on diabetes, regular foot care and recording in diary	Incidence of foot ulcers: IG*: 4.7% (n=5/NR^||^), CG^†^: 12.2% (n=14/NR^||^)
Bus, et al.^31^ 2021. Holland	n=304 (IG*: 151, CG^†^: 153) Male: 72.4% Mean age: 65 DM2^‡^: 77% Mean time since DM diagnosis: 20 years Risk of ulcer: 2/3 (IWGDF^§^)	Duration: 18 months Outcomes: Proportion of patients in each group who developed foot ulcers	IG*: Handheld infrared thermometer and recording in a standardized form developed by the researcher.	CG^†^: Foot assessment and foot screening once every 1-3 months by a podiatrist; therapeutic footwear (if indicated) and foot care education	Incidence of foot ulcers: IG*: 29.1% (n=44/151), CG^†^: 37.3% (n=57/153)
Lavery, et al.^11^ 2004. United States.	n=85 (IG*: 44, CG^†^: 41) Male: 50% Mean age: 55 years old DM2^‡^: NR Mean time since the diabetes diagnosis: 14 years Risk of ulcer: 2/3 (IWGDF^§^)	Duration: 06 months Outcomes: Proportion of patients in each group who developed foot ulcers, infections, Charcot fractures and amputations	IG*: Handheld infrared thermometer and recording in diary	GC^†^: Therapeutic footwear, foot care education, and regular assessment by a podiatrist every 10-12 weeks	Incidence of foot ulcers: IG*: 2% (n=1/44) CG^†^: 20% (n=9/41) (seven people presented ulcerations and two had Charcot arthropathies)
Lavery, et al.^17^ 2007. United States.	n=173 (IG1: 59, IG2: 56, CG: 58) Male: 54% Mean age: 65 years old DM2^‡^: 95% Mean time since the diabetes diagnosis: 13 years Risk of ulcer: 2/3 (IWGDF^§^)	Duration: 15 months Outcomes: Proportion of patients in each group who developed foot ulcers	IG1: Handheld infrared thermometer and recording in diary. IG2: Mirror for self-inspection of the feet twice a day and recording in diary	CG^†^: Evaluation of the lower limbs (physician), program, therapeutic shoes and evaluation of insoles (podiatrist), pedometer and recording in diary; inspecting the feet daily	Incidence of foot ulcers: IG1: 8.5 (n=5/59) IG2: 30.4 (n=17/56) CG^†^: 29.3 (n=17/58)
Skafjeld, et al.^28^ 2015. Norway.	n=41 (IG*: 21, CG^†^: 20) Male: 56% Mean age: 58 years old DM2^‡^: 71% Mean time since the diabetes diagnosis: 18 years Risk of ulcer: 3 (IWGDF^§^)	Duration: 12 months Outcomes: Proportion of patients in each group who developed foot ulcers	IG*: Handheld infrared thermometer, recording in diary, theory-based counseling, and pedometer for recording physical activity in the first week of the study	GC^†^: Daily foot inspection and recording in diary; use of therapeutic footwear; contacting a nurse if changes were observed	Incidence of foot ulcers: IG*: 39% (n=7/21) CG^†^: 50% (n=10/20)

*IG = Intervention Group; ^†^CG = Control Group; ^‡^DM2 = Type 2 Diabetes Mellitus; ^§^IWGDF = International Working Group on Diabetic Foot; ^||^NR = Not Reported.

### Risk of bias assessment

**Figure 3:**
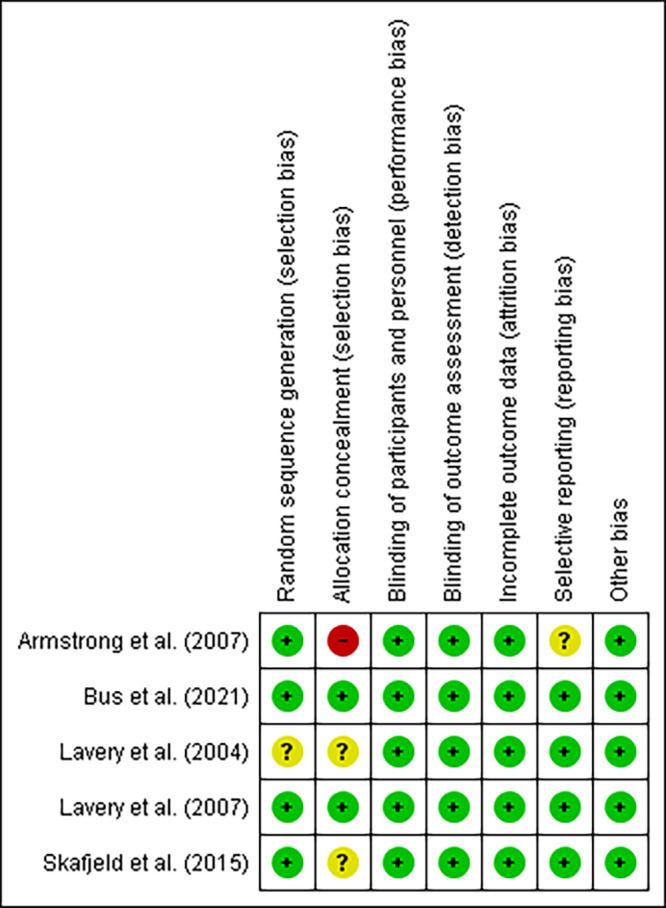
Summary of the risk of bias for the studies included

The data were analyzed in *Review Manager* 5.4^28^. As shown in Figure 3, only one study has uncertain risk of bias for “random sequence generation”^11^. Regarding the “allocation sequence”, one study presents a high risk of bias, as it does not mention the number of participants allocated to each group^16^. In turn, two of the studies have uncertain risk of bias, as they present insufficient information^11^ ^,^ ^32^. One study presented an uncertain risk of bias for the assessment of the “selective reporting” criteria^16^.

### Quantitative synthesis of the studies included: Meta-analysis

Five RCTs involving 828 participants with diabetes were identified^11^ ^,^ ^16^ ^-^ ^17^ ^,^ ^31^ ^-^ ^32^. In a study^16^, the number of participants randomized to the intervention group (thermometry) and control group (standard health care) was not mentioned and, therefore, it was not included in the meta-analysis.

**Figure 4:**
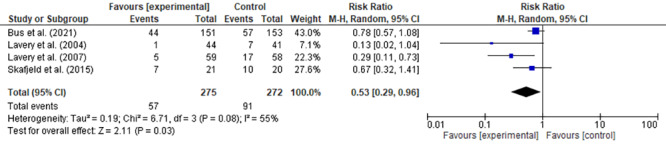
Meta-analysis of the effect of thermometry when compared to standard health care in preventing the incidence of diabetic foot ulcers

In this meta-analysis, four RCTs (n=547) were included, as shown in the forest plot graph (Figure 4), and the prevention of the incidence of diabetic foot ulcers outcome was analyzed, presented as a binary outcome. The protective effect of thermometry was evidenced when compared to standard foot care to prevent the incidence of diabetic foot ulcers (RR 0.53; 95%CI 0.29-0.96; p=0.03). The heterogeneity between studies was I²=55% (p=0.08). The number of patients needing treatment to prevent the appearance of a new ulcer was 8 (95% CI = 5-19).

### Evidence certainty classification

The Grading of Recommendations Assessment, Development and Evaluation (GRADE) system^30^ was used to assess the certainty of the evidence and results presented in the GRADE evidence profile.

Figure 5 presents an assessment for the certainty of the evidence of the meta-analysis performed according to criteria previously defined by GRADE. As already stated, the estimate of the thermometry effect to prevent the incidence of diabetic foot ulcers was RR 0.53 when compared to standard health care, supported by moderate evidence.

**Figure 5:** Classification of the evidence certainty on the effect of thermometry for the prevention of diabetic foot ulcers. GRADEpro GDT. Fortaleza, CE, Brazil, 2021

Assessment of Certainty							No. of patients		Effect		Certainty of the evidence	Importance
No. of studies	Study design	Risk of bias	Inconsistency	Indirect evidence	Imprecision	Other considerations	Skin thermometry	Standard health care	Relative (95% CI*)	Absolute (95%CI*)		
Preventing the incidence of diabetic foot ulcers (Follow-up: Mean of 12.8 months)												
4	Randomized clinical trials	Not serious	Not serious	Not serious	Serious^†^	None	57/275 (20.7%)	91/272 (33.5%)	RR^‡^ 0.53 (From 0.29 to 0.96)	16 minus by 100 (from 24 minus to 1 minus)	ꚚꚚꚚꚚО Moderate	IMPORTANT

*CI = Confidence Interval; ^†^Low number of events; ^‡^RR = Relative Risk

## Discussion

As diabetic foot ulcer represents a global public health problem, the use of preventive strategies such as foot skin thermometry can contribute in the prevention of complications inherent to this problem. However, health professionals and patients need solid evidence to implement new care models. Therefore, we conducted an SR on the effect of thermometry in DFU prevention.

Previous systematic reviews on the subject matter have already been published; however, there were inconsistencies regarding the methodological quality of the study included in the meta-analyses^20^ ^-^ ^21^. The RCT included in the meta-analyses does not specify how many individuals were allocated to the intervention group (use of thermometry) and to the control group (standard health care)^16^. Thus, failures to provide a reliable numerical summary of the intervention’s effect are observed. In addition to that, searches in data sources and comprehensive evaluation of new evidence were necessary to provide reliable and robust information.

Foot temperature was measured by the patients twice a day, in six specific regions of each foot (hallux, first, third and fifth metatarsal heads, midfoot and heel). In cases of amputations of a toe or metatarsal, temperature should be measured in an adjacent anatomical area. Temperature differences >2.2°C between the corresponding left and right sites for two consecutive days were considered to be at risk of ulceration due to inflammation at the measurement site. Thus, the patients were advised to contact the study coordinator and reduce their activity until temperature normalized. In addition to that, they were required to make diary entries about foot temperature observations.

When evaluating the outcome (incidence of foot ulcers), it was observed that in three studies there was an association of temperature monitoring with a reduction in the ulceration rate, suggesting that the thermometry used by the patients assists in the early identification of plantar inflammation before skin rupture and DFU formation^11^ ^,^ ^16^ ^-^ ^17^. On the other hand, in two studies no differences were detected between the control and intervention groups^31^ ^-^ ^32^.

In the RCT conducted by researchers from the United States, DFU incidence during a 15-month evaluation in the usual health care group was 29.3%^17^. In contrast, in the group that monitored foot temperature at the same anatomical points daily, the incidence was 8.5% (OR 4.48; 95% CI 1.53-13.14; p<0.008). 

In another study conducted by the same group of researchers, there were complications related to Charcot arthropathy (n=2)^11^. However, it is recognized that active Charcot arthropathy has an individual effect on foot skin temperature. Therefore, the data from the Charcot arthropathy cases were excluded from the analysis. Of the 84 individuals followed-up for six months, seven participants in the standard therapy group were found to have presented ulcerations. However, in the group with foot temperature monitoring, only one individual presented ulcerations (OR 8.00; 95%CI). 

Temperature measurements can be easily performed by the patients or family members and can represent an effective adjuvant in DFU prevention. In addition to that, they provide quantifiable information that shows inflammation formation in specific foot regions so that preventive measures can be taken before skin ruptures. 

It was shown that 12.2% of the 115 participants in the standard care group had ulcers and only 4.7% of the 110 individuals in the thermometry group did so^16^. Handheld thermometers can show positive results when used by high-risk patients to prevent ulcerative processes. This may offer an additional advantage to conventional DFU prevention practices and therapies. 

Half of the 10 people in the standard care group presented ulcerations and 39% of the 21 individuals in the temperature monitoring group presented the same outcome, with no significant intergroup differences (p=0.532)^32^. Although there were no intergroup differences in DFU recurrence, thermometry proved to be feasible for the patients. 

In the largest RCT on the topic up to date, 44 of 151 (29.1%) who used thermometry had a recurrent ulcer at a primary site, which was not significantly different from the 57 of 153 (37.3%) participants in the usual care group (RR 0.782; 95% CI 0.566-1.080; p=0.133). However, when the participants reduced their activity when an inflammation point was identified, the intervention proved to be effective over usual care^31^. 

On the other hand, ensuring that the patients use the thermometer daily at their homes and refrain from all daily activities when the temperature of their feet is high is a potential challenge. Moreover, in the real world, adherence to the device for long periods of time can be lower than those recorded in this study.

The summary of the effect of the intervention points to a reduction in the number of ulcers in the group using foot skin thermometry, when compared to the standard care group (RR=0.53; 95%CI 0.29-0.96; I²=55%; p=0.08). Although statistical heterogeneity is substantial (I²=55%), it should be noted that it is not statistically significant. 

The number of patients needed to be treated to prevent the appearance of a new skin ulcer was 8. This metric has been valuable in the clinical practice, especially in the selection of therapeutic interventions. It also has the potential to be used as a support tool in risk-benefit assessments and to help in health-related decision-making^33^. Thus, skin thermometry represents an intervention that is easy to apply and its preventive role can contribute to the reduction of the high health costs and serious complications, such as hospitalizations, amputations and deaths. 

Estimating the efficacy of using thermometry in DFU prevention is supported by certainty of moderate evidence. Presence of imprecision was identified. It is recommended to lower the certainty of the evidence when the CI overlaps the null done line or the total number of events is less than 300. It should be noted that the estimates from the four studies included favor the intervention and that there is some overlap in the confidence intervals. In this case, it is not justified to lower the certainty of the evidence. However, the number of events is far from the recommended “optimal information size”^34^. 

The strengths of this SR include explicit definition of the inclusion and exclusion criteria for the studies, use of a comprehensive and highly sensitive search strategy, rigorous screening and adherence to the PRISMA checklist, reliable numerical summary of the intervention’s effect, use of methodological quality assessment tools and supplementation with a GRADE evidence certainty assessment. This systematic and sensitive search made it possible to group interventions with similar characteristics. 

The treatment of diabetic foot ulcer complications and consequent lower limb amputations is always more expensive than investing in preventive measures and approaches to the foot at risk of ulceration. Therefore, thermal monitoring of the feet in people with diabetes has the potential to contribute to Clinical Nursing and to the interprofessional practice, by identifying imminent ulcerative processes, preventing new injuries, avoiding lower limb amputations and improving the individual’s quality of life, in addition to being able to reduce the burden on the health services and public expenditures. 

In this context, the implementation of skin thermometry assessment in the clinical protocol for the examination of individuals with diabetes is presented as a potential approach, due to its effectiveness in preventing ulcerations, in addition to being a non-invasive technique, requiring a short screening time, and with viable applicability. In addition, daily self-monitoring of dermal temperature by the patients or with the help of family members/caregivers can prevent occurrence and recurrence of diabetic foot ulcers.

One of the limitations found in this research was that few studies met the inclusion criteria for this systematic review. The authors of this review contacted the authors of the studies included via the researchers’ digital platform (https://www.researchgate.net/) to solve doubts, but received no answers. 

It is recommended that future research studies with larger samples be carried out to evaluate the use of this intervention; as well as that the cost of foot thermal monitoring in people with Diabetes Mellitus be also evaluated, via multicenter research studies in multiple social contexts. It is relevant to consider whether this intervention is profitable to a wider population in health services or at their homes. It is believed that implementing this new preventive approach can stand out in relation to the high financial costs with complications of diabetic plantar ulcers and lower limb amputations.

New research studies on thermometry as a tool for the thermal assessment of the feet in people with diabetes are underway, and they can be followed-up via the https://www.clinicaltrials.gov/.

## Conclusion

It was evidenced that the use of infrared thermometers to monitor plantar temperature is a promising tool in the prevention of foot ulcers in people with Diabetes Mellitus. It is hoped that the findings of this systematic review with meta-analysis will sensitize and encourage managers, public health services, health professionals and patients/family members/caregivers to implement this preventive technique in the clinical and home contexts, as diabetic foot ulcers represent a high burden for the global public health.

Incorporating this new preventive approach has the potential to contribute to the promotion of interdisciplinary and interprofessional care for the health team, in addition to promoting clinical decision-making, in conjunction with the patient’s wishes, improving their health conditions and contributing to the population and managers, by allowing planning, organization and reinforcement of new preventive strategies.

## References

[1] International Working Group on the Diabetic Foot (2019). IWGDF Guidelines on the prevention and management of diabetic foot disease.

[2] Selvarajah D, Kar D, Khunti K, Davies MJ, Scott AR, Walker J (2019). Diabetic peripheral neuropathy: advances in diagnosis and strategies for screening and early intervention. Lancet Diabetes Endocrinol.

[3] Tchero H, Kangambega P, Lin L, Mukisi-Mukaza M, Brunet-Houdard S, Briatte C (2018). Cost of diabetic foot in France, Spain, Italy, Germany and United Kingdom: A systematic review. Ann Endocrinol (Paris).

[4] Monteiro-Soares M, Russell D, Boyko EJ, Jeffcoate W, Mills JL, Morbach S (2020). Guidelines on the classification of diabetic foot ulcers (IWGDF 2019). Diabetes Metab Res Rev.

[5] Armstrong DG, Lavery LA (1996). Monitoring neuropathic ulcer healing with infrared dermal thermometry. J Foot Ankle Surg.

[6] Armstrong DG, Lavery LA, Wunderlich RP, Boulton AJM (2003). Skin Temperatures as a One-time Screening Tool Do Not Predict Future Diabetic Foot Complications. J Am Podiatr Med Assoc.

[7] Armstrong DG, Lipsky BA, Polis AB, Abramson MA (2006). Does dermal thermometry predict clinical outcome in diabetic foot infection? Analysis of data from the SIDESTEP trial. Int Wound J.

[8] Wukich DK, Raspovic KM, Suder NC (2018). Patients With Diabetic Foot Disease Fear Major Lower-Extremity Amputation More Than Death. Foot Ankle Spec.

[9] Martins-Mendes D, Monteiro-Soares M, Boyko EJ, Ribeiro M, Barata P, Lima J (2014). The independent contribution of diabetic foot ulcer on lower extremity amputation and mortality risk. J Diabetes Complications.

[10] Sibbald RG, Mufti A, Armstrong DG (2015). Infrared Skin Thermometry. Adv Skin Wound Care.

[11] Lavery LA, Higgins KR, Lanctot DR, Constantinides GP, Zamorano RG, Armstrong DG (2004). Home Monitoring of Foot Skin Temperatures to Prevent Ulceration. Diabetes Care.

[12] Goller H, Lewis DW, Mclaughlin RE (1971). Thermographic studies of human skin subjected to localized pressure. Am J Roentgenol.

[13] Sandrow RE, Torg JS, Lapayowker MS, Resnick EJ (1972). The Use of Thermography in the Early Diagnosis of Neuropathic Arthropathy in the Feet of Diabetics. Clin Orthop Relat Res.

[14] Stess RM, Sisney PC, Moss KM, Graf PM, Louie KS, Gooding GAW (1986). Use of Liquid Crystal Thermography in the Evaluation of the Diabetic Foot. Diabetes Care.

[15] Armstrong DG, Lavery LA (1997). Monitoring healing of acute Charcot's arthropathy with infrared dermal thermometry. J Rehabil Res Dev.

[16] Armstrong DG, Holtz-Neiderer K, Wendel C, Mohler MJ, Kimbriel HR, Lavery LA (2007). Skin Temperature Monitoring Reduces the Risk for Diabetic Foot Ulceration in High-risk Patients. Am J Med.

[17] Lavery LA, Higgins KR, Lanctot DR, Constantinides GP, Zamorano RG, Athanasiou KA (2007). Preventing Diabetic Foot Ulcer Recurrence in High-Risk Patients: Use of temperature monitoring as a self-assessment tool. Diabetes Care.

[18] Lazo-Porras M, Bernabe-Ortiz A, Taype-Rondan A, Gilman RH, Malaga G, Manrique H (2020). Foot thermometry with mHeath-based supplementation to prevent diabetic foot ulcers: A randomized controlled trial. Wellcome Open Res.

[19] Crawford F, Nicolson DJ, Amanna AE, Martin A, Gupta S, Leese GP (2020). Preventing foot ulceration in diabetes: systematic review and meta-analyses of RCT data. Diabetologia.

[20] Ena J, Carretero-Gomez J, Arevalo-Lorido JC, Sanchez-Ardila C, Zapatero-Gaviria A, Gómez-Huelgas R (2021). The Association Between Elevated Foot Skin Temperature and the Incidence of Diabetic Foot Ulcers: A Meta-Analysis. Int J Low Extrem Wounds.

[21] Alahakoon C, Fernando M, Galappaththy C, Matthews EO, Lazzarini P, Moxon JV (2020). Meta-analyses of randomized controlled trials reporting the effect of home foot temperature monitoring, patient education or offloading footwear on the incidence of diabetes-related foot ulcers. Diabet Med.

[22] Page MJ, McKenzie JE, Bossuyt PM, Boutron I, Hoffmann TC, Mulrow CD (2021). The PRISMA 2020 statement: an updated guideline for reporting systematic reviews. BMJ.

[23] Higgins JPT, Thomas J, Chandler J, Cumpston M, Li T, Page MJ (2021). Cochrane Handbook for Systematic Reviews of Interventions version 6.2 (updated February 2021).

[24] Schardt C, Adams MB, Owens T, Keitz S, Fontelo P (2007). Utilization of the PICO framework to improve searching PubMed for clinical questions. BMC Med Inform Decis Mak.

[25] Siddaway AP, Wood AM, Hedges LV (2019). How to Do a Systematic Review: A Best Practice Guide for Conducting and Reporting Narrative Reviews, Meta-Analyses, and Meta-Syntheses. Annu Rev Psychol.

[26] Ouzzani M, Hammady H, Fedorowicz Z, Elmagarmid A (2016). Rayyan-a web and mobile app for systematic reviews. Syst Rev.

[27] Sterne JAC, Savovic J, Page MJ, Elbers RG, Blencowe NS, Boutron I (2019). RoB 2: a revised tool for assessing risk of bias in randomised trials. BMJ.

[28] The Cochrane Collaboration (2020). Review Manager (RevMan).

[29] Higgins JPT (2003). Measuring inconsistency in meta-analyses. BMJ.

[30] Guyatt GH, Oxman AD, Vist GE, Kunz R, Falck-Ytter Y, Alonso-Coello P (2008). GRADE: an emerging consensus on rating quality of evidence and strength of recommendations. BMJ.

[31] Bus SA, Stegge WB, Baal JG, Busch-Westbroek TE, Nollet F, Netten JJ (2021). Effectiveness of at-home skin temperature monitoring in reducing the incidence of foot ulcer recurrence in people with diabetes: a multicenter randomized controlled trial (DIATEMP). BMJ Open Diabetes Res Care.

[32] Skafjeld A, Iversen MM, Holme I, Ribu L, Hvaal K, Kilhovd BK (2015). A pilot study testing the feasibility of skin temperature monitoring to reduce recurrent foot ulcers in patients with diabetes - a randomized controlled trial. BMC Endocr Disord.

[33] Mendes D, Alves C, Batel-Marques F (2017). Number needed to treat (NNT) in clinical literature an appraisal. BMC Med.

[34] Guyatt GH, Oxman AD, Kunz R, Brozek J, Alonso-Coello P, Rind D (2011). GRADE guidelines 6 Rating the quality of evidence-imprecision. J Clin Epidemiol.

